# Bismuth Redox Catalysis: An Emerging Main-Group Platform for Organic Synthesis

**DOI:** 10.1021/acscatal.1c04897

**Published:** 2022-01-07

**Authors:** Hye Won Moon, Josep Cornella

**Affiliations:** Max-Planck-Institut für Kohlenforschung, Kaiser-Wilhelm-Platz 1, Mülheim an der Ruhr, 45470, Germany

**Keywords:** Main group catalysis, redox cycling, organobismuth, organic synthesis, heavy elements

## Abstract

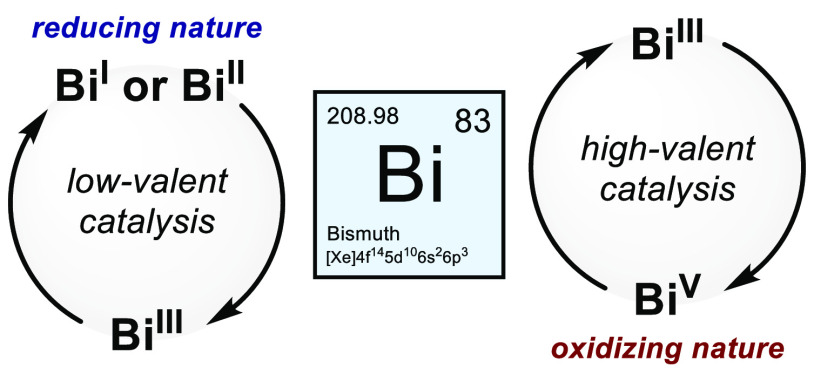

Bismuth has recently been shown to be able to maneuver between different oxidation states, enabling access to unique redox cycles that can be harnessed in the context of organic synthesis. Indeed, various catalytic Bi redox platforms have been discovered and revealed emerging opportunities in the field of main group redox catalysis. The goal of this perspective is to provide an overview of the synthetic methodologies that have been developed to date, which capitalize on the Bi redox cycling. Recent catalytic methods via low-valent Bi(II)/Bi(III), Bi(I)/Bi(III), and high-valent Bi(III)/Bi(V) redox couples are covered as well as their underlying mechanisms and key intermediates. In addition, we illustrate different design strategies stabilizing low-valent and high-valent bismuth species, and highlight the characteristic reactivity of bismuth complexes, compared to the lighter *p*-block and *d*-block elements. Although it is not redox catalysis in nature, we also discuss a recent example of non-Lewis acid, redox-neutral Bi(III) catalysis proceeding through catalytic organometallic steps. We close by discussing opportunities and future directions in this emerging field of catalysis. We hope that this Perspective will provide synthetic chemists with guiding principles for the future development of catalytic transformations employing bismuth.

## 1 Introduction

Catalysis based on transition metals (TMs) has undoubtedly had a profound impact in synthetic chemistry, ranging from laboratory routes to industrial-scale processes.^1,2^ One of the key underlying principles for the success of TM catalysis lies on their facile redox cycling between different oxidation states, which is coupled with bond formation or cleavage in its coordination sphere.^3,4^ For decades, such redox properties were believed to be the realm of TMs, and elements beyond the *d*-block were certainly not considered for such purposes. However, such dogmatic belief has been recently challenged, as many main group elements have been demonstrated to undergo elementary redox reactions that were once believed to be reserved for TMs. It is now common to see a growing number of articles in which *s*- and *p*-block elements undergo oxidative addition, reductive elimination, etc., leading to reactivity that resembles that of TMs and, in some instances, complements them.^5−8^ Despite the wealth of literature on the development of stoichiometric redox reactivity by main group elements, the development of efficient catalytic redox processes is challenging and is still in its infancy.

**Figure 1:**
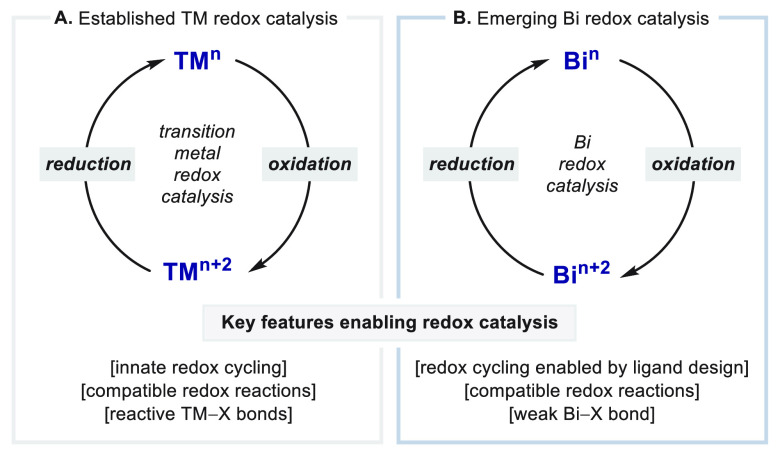
Analogy between (A) the established field of transition-metal (TM) redox catalysis and (B) a new area of catalysis using Bi complexes.

A major challenge is that unlocking catalytic redox cycle in main group elements requires a complete redesign of the approach when compared to TMs, because of the different electronic situation encountered for such elements (Figure 1). Generally, the facile access to various stable oxidation states in TM complexes is a consequence of the small energy gap between their frontier *d*-orbitals. On the other hand, the gap between the frontier *s*-/*p*-based orbitals in *p*-block elements is substantially larger.^5^ As a result, redox events at *p*-block elements generally occur unidirectionally in a stoichiometric fashion, facilitated by strong thermodynamic driving forces. Hence, the *p*-block products in the redox reactions are often thermodynamic sinks, and their recycling toward catalytic protocols becomes problematic. For example, oxidative addition of E–H or C–X (E = H, C, N, O; X = F, Cl, Br, I) bonds to low-valent group 13 or 14 compounds is often favorable,^9−12^ but reductive elimination from the high-valent compounds of these elements is challenging. In contrast, C–H, C–C, or C–X (X = F, Cl) bond formation via reductive elimination from high-valent group 16 or 17 compounds is well-established;^13,14^ yet, oxidative addition to low-valent compounds of these elements is rare. Because of such biased thermodynamics between low-valent and high-valent species, merging redox elementary steps into redox cycling remains a tremendous challenge for the aforementioned *p*-block elements.

The group 15 elements, or pnictogens, are located in a transitional position on the *p*-block from a reactivity standpoint. Hence, a bidirectional Pn(*n*)/Pn(*n*+2) redox couple at group 15 elements should be viable.^15,16^ Considering the foregoing pnictogen properties, the Radosevich group pioneered phosphorus redox catalysis via a P(III)/P(V) cycle.^17^ By virtue of strained tridentate and bidentate ligands around phosphorus, the group overcame the large energetic gap between the frontier orbitals, thereby enabling redox catalysis. In addition, by lowering the kinetic barrier of phosphine oxide reduction, numerous deoxygenative transformations via P(III)/P(V)=O cycling have been accomplished.^16,18^ While the heavier congeners, arsenic and antimony, have a few examples of redox catalysis,^19,20^ the heaviest stable element in group 15—bismuth—has had limited applications until recently, despite its low cost and low toxicity.^21^ Indeed, bismuth has presented its candidacy as a versatile element in redox catalysis, given its rich redox chemistry and labile bonding nature that can enable new organic transformations and small molecule activation through Bi redox cycling.

Traditionally, the catalytic utility of Bi(III) compounds in synthesis was limited to nonredox soft Lewis acid catalysis such as carbonyl and diene activation.^22−24^ Organobismuth(III) compounds also found synthetic applications as transmetalation reagents, because of the lability of Bi(III)–C bonds.^25^ In the presence of strong oxidants, these compounds undergo oxidation leading to organobismuth(V) compounds, which have been applied as oxidants in synthesis.^26^ Moreover, the labile nature of the Bi(V)–C bond has also been employed in several stoichiometric oxidative ligand coupling events toward C–C or C–X bond formation.^27,28^ In heterogeneous electrocatalysis or photocatalysis, Bi is frequently encountered as a dopant in materials in order to advance performance (e.g., selectivity and efficiency) in CO_2_ reduction, water splitting, and dinitrogen reduction.^29^ Despite these seminal reports indicating the rich redox chemistry of bismuth, the development of catalytic redox processes for organic synthesis have been largely overlooked. In this Perspective, we describe how the area of *bismuth redox catalysis* has flourished with illustration of recent developments in Bi(II)/Bi(III), Bi(I)/Bi(III), and Bi(III)/Bi(V) redox cycles. We aim to show design principles for different redox cycles toward new catalytic transformations. Albeit not via redox cycling, we also provide an example of redox-neutral Bi(III) catalysis via elementary organometallic steps. We believe that this Perspective provides a summary of the unique reactivity that makes bismuth a versatile element for redox catalysis, with properties that go beyond a mere TM alternative.

## 2 Low-Valent Catalysis

Unlike the lighter pnictogen elements, Bi has been shown to expand beyond the canonical Pn(III)/Pn(V) redox pair (see Section 3), enabling redox cycling at a rather unique low-valent platform, and hence, explore its reductive properties.^30^ In this section, we describe Bi(II)/Bi(III) and Bi(I)/Bi(III) redox cycles and the organic transformations derived thereof.

### 2.1 Low-Valent Bi(II)/Bi(III) Catalysis

The current examples of other group 15 elements in redox catalysis capitalize on the two-electron Pn(*n*)/Pn(*n*+2) redox pair for productive catalysis. In contrast, bismuth offers a unique opportunity for reactivity beyond such canonical redox pair through one-electron Bi(II)/Bi(III) redox processes.

**Figure 2:**
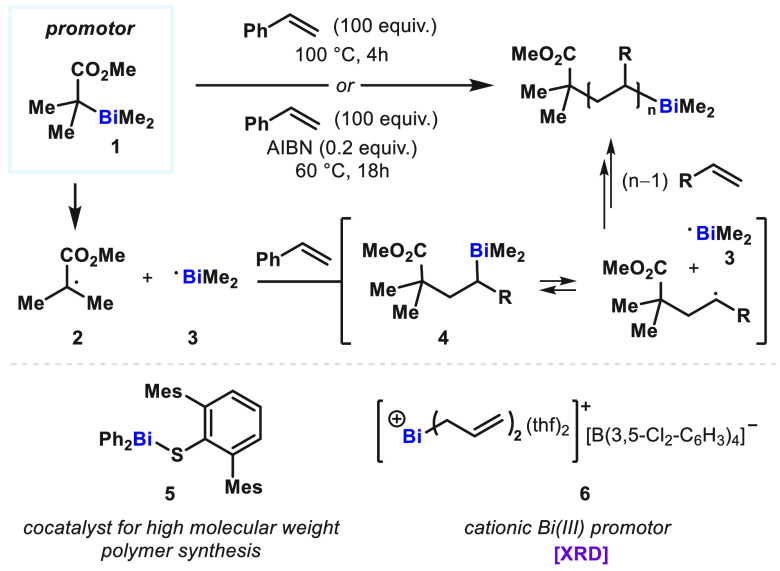
Bismuth-mediated radical polymerization via a putative Bi(II) radical intermediate.

As a seminal example of Bi(II) radical intermediates for synthetic use, Yamago reported organobismuthine-mediated radical polymerization reactions in 2007 (Figure 2).^31^ It was proposed that a bismuthine promotor **1** undergoes Bi–C bond homolysis to generate alkyl radical **2** and Bi(II) radical **3** through thermal decomposition or radical initiation by a substoichiometric amount of 2,2′-azobis(isobutyronitrile) (AIBN). The resulting Bi(II) species **3** promotes radical polymerization via the intermediacy of alkylbismuthine **4** that can further promote polymerization through homolytic Bi–C cleavage, enabling highly controlled living radical polymerization. In a follow-up report in 2009,^32^ arylthiobismuthine **5** is used as a co-catalyst for the synthesis of high-molecular-weight polystyrenes (*M*_n_ = 198 000 in the presence of **5**; *M*_n_ = 107 000 in the absence of **5**), given that thiyl radicals facilitate the homolytic substitution by alkyl radicals, as previously described by Barton.^33^ A cationic Bi(III) species **6** was reported by Lichtenberg and Okuda, which was also shown to drive the radical polymerization of styrene via Bi–C homolysis.^34^

**Figure 3:**
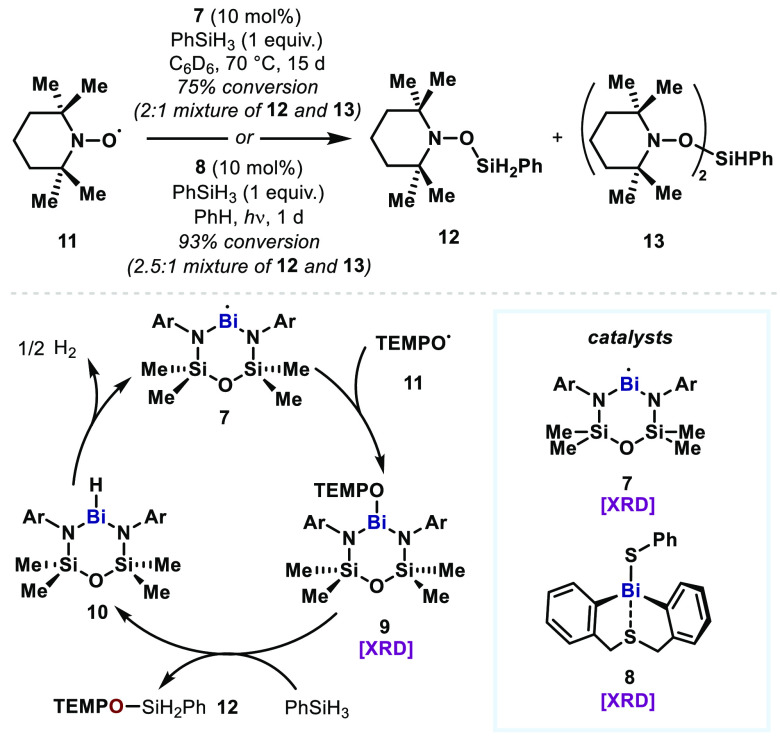
Bismuth-catalyzed dehydrocoupling of TEMPO and hydrosilanes via a Bi(II)/Bi(III) redox cycle.

While transient Bi(II) species have been proposed in polymerization reactions, in 2014, Iwamoto first reported a persistent Bi(II) radical that is in equilibrium with its dimer in solution.^35^ Later, Coles reported the first monomeric Bi(II) species **7** that is stable both in solution and in the solid state (Figure 3).^36^ Building on this seminal work on the isolable Bi(II) species, in 2018, Coles reported a Bi(II)/Bi(III) redox cycle in a catalytic dehydrocoupling reaction of TEMPO radical (**11**) and phenylsilane using Bi(II) complex **7** as a catalyst.^37^ This stable and isolable complex (**7**) supported by bis(amido)disiloxane ligand was previously shown to exhibit one-electron reducing character in a stoichiometric manner.^38^ Merging its reducing character and the reactive nature of Bi(III)–O bonds,^39^ the catalytic dehydrocoupling of TEMPO (**11**) and phenylsilane was achieved, albeit under high temperatures and long reaction times. In this reaction, **7** undergoes radical recombination with TEMPO (**11**) to generate Bi(III)–TEMPOxide intermediate **9**. This resulting Bi(III) species is then proposed to undergo metathesis with the Si–H bond in phenylsilane to produce the dehydrocoupling product **12** and a Bi(III)–hydride species **10**. The putative Bi(III)–hydride species **10** was previously shown to oxidatively generate H_2_,^38b^ which would regenerate the Bi(II) catalyst **7** and close the Bi(II)/Bi(III) redox cycle.

An analogous Bi(II)/Bi(III) cycle was demonstrated by Lichtenberg using a diaryl(bismuth)thiolate precatalyst **8**.^40^ It is proposed to generate a transient Bi(II) species upon UV irradiation via Bi–S bond homolysis and initiate the aforementioned Bi(II)/Bi(III) cycle toward dehydrocoupling. The comparatively faster reaction rates were achieved by in situ generation of the presumed catalytically active Bi(II) species.

**Figure 4:**
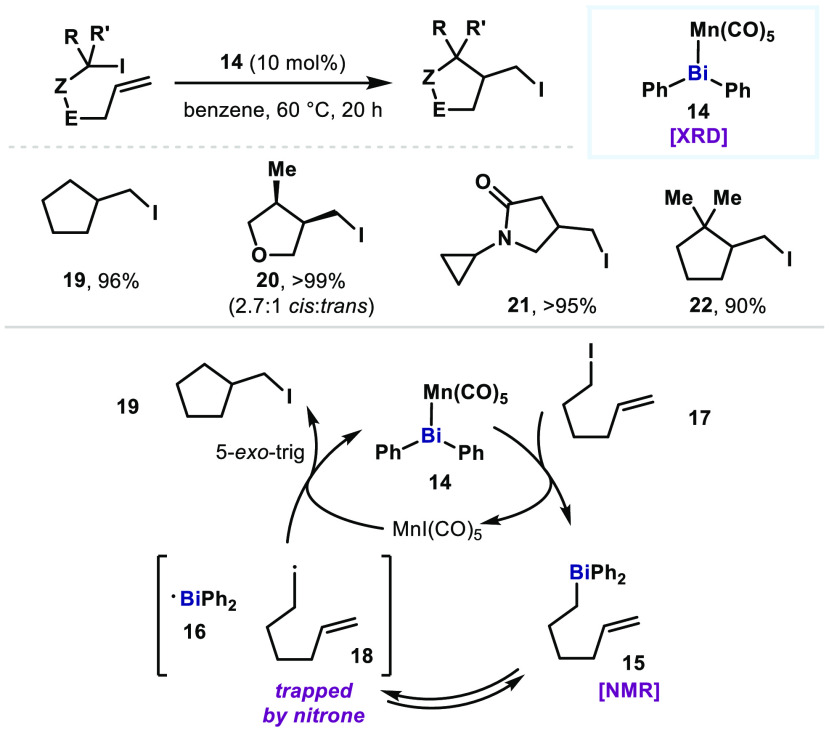
Cycloisomerization of δ-iodo-olefins using a TM bismuthane catalyst.

In 2019, Lichtenberg also demonstrated a radical cycloisomerization of δ-iodo-olefins using a TM bismuthane **14**, which would potentially proceed via a Bi(II)/Bi(III) cycle (Figure 4).^41^ It was shown that δ-iodo-olefins were readily cyclized to form 5-membered rings (**19**–**22**). In the reaction mixture, MnI(CO)_5_ and alkyldiphenylbismuth compound **15** were detected by IR and NMR spectroscopy, respectively. The Bi(III) compound **15** would potentially undergo Bi–C homolysis to generate Bi(II) species **16** and alkyl radical **18**, and ultimately deliver **19** through a 5-*exo*-trig cyclization and radical trap by MnI(CO)_5_ or **17**. In the presence of nitrone, the alkyl radical **18** can be trapped, supporting the proposed radical mechanism. Although complex **14** can behave both as a redox catalyst or a radical initiator, such radical chemistry is successfully triggered thermally because of the weak Bi–Mn bond.

### 2.2 Low-Valent Bi(I)/Bi(III) Catalysis

**Figure 5:**
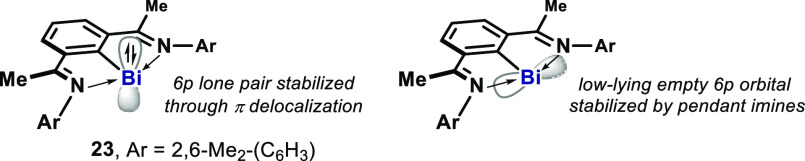
First stable monomeric *N*,*C*,*N*-chelated bismuthinidene and the Bi valence orbitals.

While the 6*s*^2^ nonbonding lone pair in Bi(III) compounds is relatively low in energy and rather inert,^42^ the 6*p*^2^ lone pair in Bi(I) compounds has been shown to be nucleophilic and readily oxidized. Since the seminal report by Tokitoh in 1997, organobismuth(I) compounds have largely been reported as oligomeric or dimeric structures.^43^ It was not until 2010, when Dostál reported the first monomeric, well-defined organobismuth(I) complex supported by an *N,C,N*-pincer ligand (Figure 5).^44a^ Access to this isolable bismuthinidene **23** is feasible due to its stability rendered by the two N atoms in the imine arms, which coordinate to the low-lying Bi empty *p*-orbital.^44b^ In addition, the 6*p*^2^ lone pair in the *p*_*z*_ orbital perpendicular to the ligand plane, is stabilized further through delocalization across the π-bond of the ligand.

**Figure 6:**
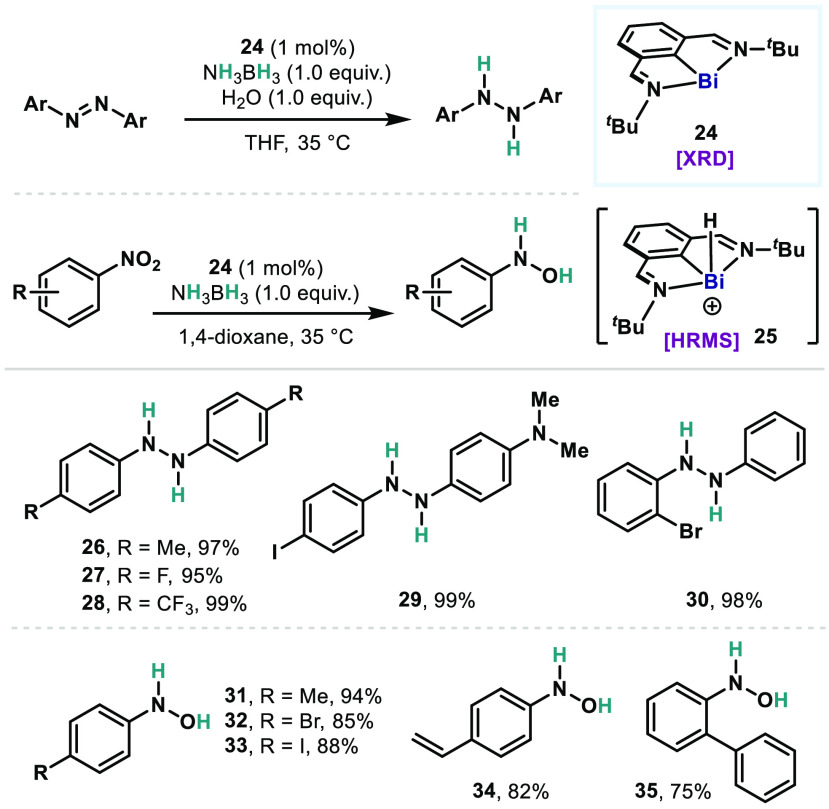
Bismuth-catalyzed transfer hydrogenation of azoarenes and nitroarenes to diarylhydrazines and *N*-arylhydroxylamines, respectively, with ammonia borane.

Building upon Dostál’s complex (**23**), in 2019, our group demonstrated that an analogous Bi(I) complex **24** is a competent catalyst in the context of transfer hydrogenation (Figure 6).^45^ Under the optimized reaction conditions, the complex **24** catalyzes the transfer hydrogenation from ammonia borane to either azoarenes or nitroarenes across the N–N or N–O π-bond, respectively, with good functional group tolerance. Azoarenes containing electron-donating or withdrawing groups can be reduced to the corresponding hydrazines in good yields (**26**–**28**), and an unsymmetrical azoarene in a push–pull situation was also well-tolerated (**29**). A bromide substituent on the *ortho*-position did not affect the reactivity (**30**). Analogously, nitroarenes were reduced into the corresponding *N*-hydroxylamines in good yields, which complements TM-catalyzed reduction reactions.^46^ Electron-donating group (**31**), halide substituents (**32**, **33**), and unsaturated functionality (**34**) were well-tolerated. 2-Phenylnitrobenzene was selectively reduced to the *N*-hydroxylamine **35** without formation of carbazole, which is complementary to the Cadogan-type P(III) catalysis.^47^ In analogy with the intermediacy of the P(V)-dihydride in the previously described transfer hydrogenation via a P(III)/P(V) cycle, it is proposed that this catalytic cycle also involves the intermediacy of organobismuth(III) hydrides, such as **25**, that can be detected by HRMS in the reaction mixture.^17a^ However, the detailed mechanism including Bi–H bond formation/cleavage and N–H bond formation in the products is yet unknown.

**Figure 7:**
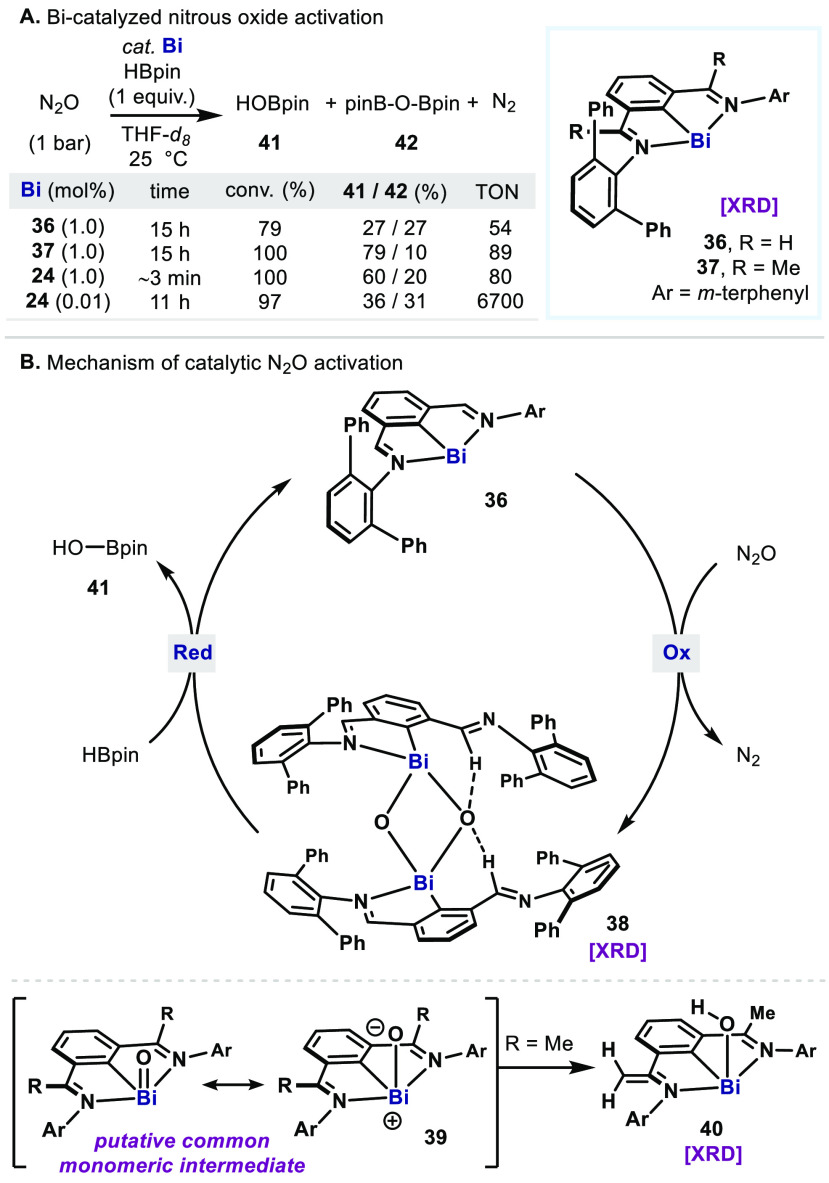
(A) Bismuth-catalyzed deoxygenation of nitrous oxide with pinacol borane. (B) Proposed mechanism via the intermediacy of bismuth oxides.

Another example of the low-valent Bi(I)/Bi(III) redox platform can be found in the catalytic activation of nitrous oxide (N_2_O) (Figure 7A).^48^ Exposure of the Bi catalyst **24**, used in the transfer hydrogenation, to N_2_O atmosphere (1 bar) resulted in the rapid liberation of N_2_ and the formation of a putative dimeric bismuth oxide species. The resulting oxo species can be detected by HRMS—also observed in related bismuth oxo or sulfido dimer precedents^49^—but it could not be isolated or crystallographically characterized because of its thermal instability. In an attempt to characterize the organobismuth(III) intermediates, two new Bi(I) complexes were synthesized which were supported by aldimines (**36**) and ketimines (**37**) bearing *m*-terphenyl substituents.^50^ Upon exposure to N_2_O, the complex **36** afforded a dimeric bismuth oxide **38** with a [Bi_2_O_2_] core as its *anti-*isomer which was identified by single-crystal X-ray crystallography (Figure 7B). By contrast, the ketimine-supported **37** gave a monomeric bismuth hydroxide (**40**), presumably as a result of deprotonation of one of the Me groups in the backbone. These observations suggest the involvement of a transient monomeric bismuth oxide (**39**) upon *O*-atom abstraction from N_2_O, which subsequently undergoes dimerization or deprotonation via the highly polarized Bi=O bond. Eventually, treating complexes **38** and **40** with pinacolborane (HBpin, 2 equiv) resulted in regeneration of **36** and **37**, respectively, and formation of the mixture of HOBpin (**41**) and O(Bpin)_2_ (**42**).

With validated oxidation and reduction as potential catalytic elementary steps, catalytic reactions were performed using these Bi(I) catalysts **36**, **37**, and **24**. Indeed, the catalysts drive N_2_O deoxygenation with TON of 54, 89, and 80, respectively. The most reactive **24** toward N_2_O enabled lowering the catalyst loading to 0.01 mol % and enhancing the TON to 6700 (TOF ≈ 52 min^–1^). This is an unprecedented catalytic activation of N_2_O by main group elements,^51−53^ which performs comparable or beyond TM deoxygenation catalysis.^54−57^ Albeit restricted to HBpin, this work reveals the potential of Bi catalysts for *O*-atom transfer (OAT), which can eventually be applied to organic substrates in catalytic oxidative transformations.

**Figure 8:**
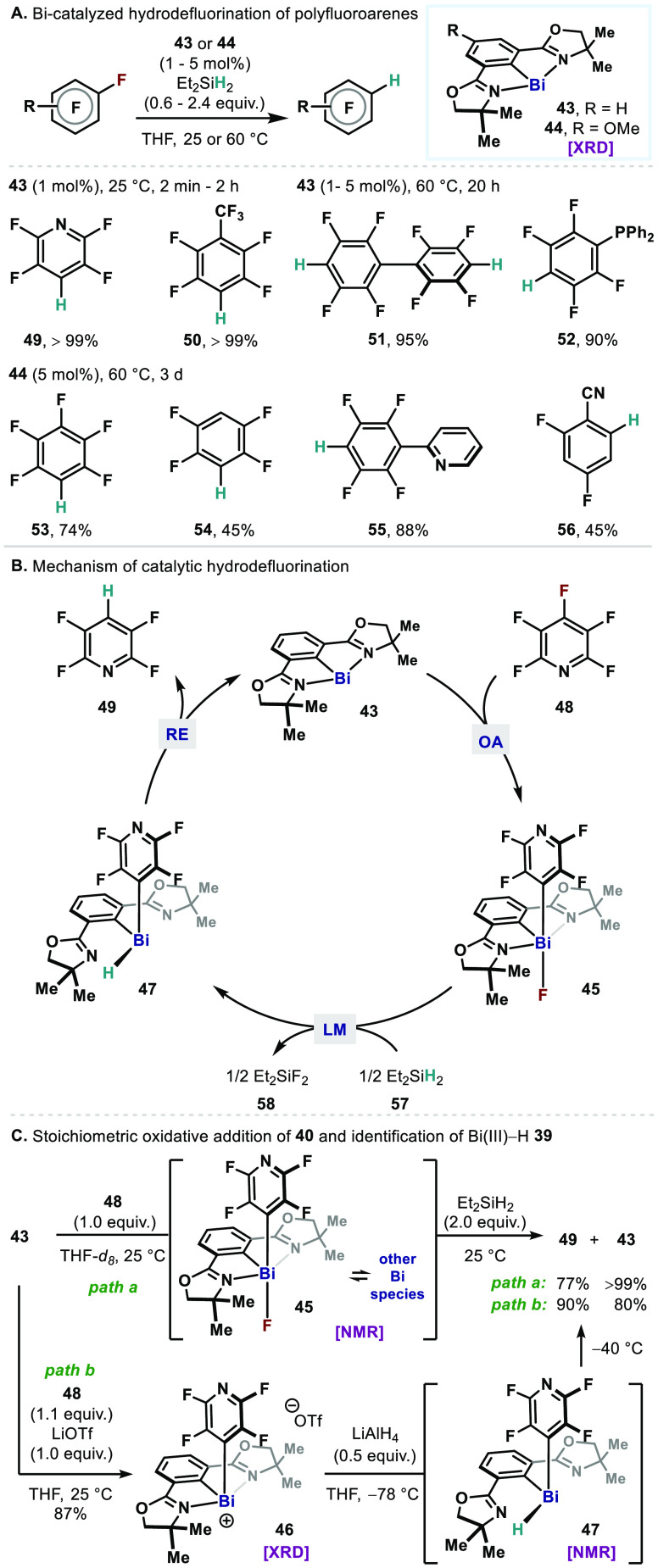
(A) Bismuth-catalyzed hydrodefluorination (HDF) of polyfluoroarenes with diethylsilane. (B) Proposed mechanism of HDF. (C) Validation of C–F oxidative addition and C–H reductive elimination at the Bi center.

The catalytic utility of the *N*,*C*,*N*-chelated bismuthinidenes via a Bi(I)/Bi(III) redox cycle has been further expanded for hydrodefluorination (HDF) (Figure 8A).^58^ In this case, new bismuthinidenes **43** and **44** are employed, featuring oxazolines as pendant supporting arms, which is distinct from the previously reported Bi(I) complexes (i.e., **24**, **36**, **37**). While HDF of hexafluorobenzene using the catalyst **24** (5 mol %) gave a trace amount of the product (**53**) in the presence of diethylsilane (2.4 equiv), the use of the newly designed catalysts **43** and **44** supported by a 2,6-bis(oxazolinyl)phenyl (Phebox) scaffold resulted in improvement to 40% and 74% yields, respectively. The structure of **43** and **44** that contains elongated Bi–C bonds and longer Bi–N bonds indicates that the advanced reactivity is achieved by virtue of more localized 6*p*_*z*_ lone pair orbital in **43** and **44** compared to that in complex **24** (Bi–C: 2.193(6) Å for **43**, 2.201(4) Å for **44**, 2.146(18) Å for **24**; Bi–N: 2.502(3) and 2.525(3) Å for **43**, 2.5142(18) and 2.5359(19) Å for **44**, 2.492(8) and 2.502(9) Å for **24**). This method hydrodefluorinates a variety of polyfluoroarenes under mild conditions. Pentafluoropyridine and pentafluorobenzene with a strong electron-withdrawing group underwent HDF to give the products (**49**, **50**) in quantitative yields at ambient temperature using **43**. Di-HDF (**43**) and HDF (**44**) of highly fluorinated phosphine are viable at 60 °C. Partially fluorinated substrates can be defluorinated (**54**–**56**), using **44** as a catalyst in moderate yields. No directing effect was observed (**55**), providing orthogonal selectivity to TM catalysis.^59^ Polyfluoroarenes bearing an electron-donating group were found to be challenging, as similarly observed in HDFs using TM catalysts.^60^ Mechanistically, it is proposed that the Bi(I) catalyst **43** undergoes intermolecular oxidative addition (OA) of C(sp^2^)–F bond to give fluoroarylbismuthine **45** (Figure 8B). Subsequent ligand metathesis (LM) with diethylsilane (**57**) leads to the formation of hydridoarylbismuthine **47**. It was found that a substoichiometric amount of **57** can be used as a 2 equiv hydride source, giving diethylfluorosilane (**58**) upon completion of the reactions. Lastly, reductive elimination (RE) of C–H bond gives the HDF product **49** and regenerates the Bi(I) catalyst **43** to close the catalytic redox loop.

Each elementary step of the cycle was studied and validated experimentally (Figure 8C). In the stoichiometric reaction with pentafluoropyridine (**48**), it was shown that Bi(I) complex **43** undergoes C–F OA to give a fluoroarylbismuthine **45** (Figure 8C, path a). It was observed that bismuthine **45** interconverts with other Bi species in the reaction mixture, but it could be characterized by multinuclear one-dimensional (1D) and two-dimensional (2D) nuclearmagnetic resonance (NMR) spectroscopy. Subsequent addition of diethylsilane to this mixture resulted in the formation of HDF product **49** and regeneration of Bi(I) complex **43**. It was hypothesized that the labile Bi–F bond in **45** is attributed to the observed interconversion, and the isolation of cationic Bi(III) species **46** was attempted using LiOTf (Figure 8C, path b). Indeed, a stable cationic Bi(III) complex **46** was obtained, which was characterized by single-crystal X-ray crystallography. Upon treating **46** with LiAlH_4_ (0.5 equiv), the formation of hydridoarylbismuthine **47** was observed, which at elevated temperature, gave HDF product **49** and the Bi(I) complex **43**. These three elementary steps, OA, LM, and RE, are akin to those in a well-defined P(III)/P(V) synthetic cycle for HDF previously demonstrated by Radosevich.^61^ While a catalytic OA/LM/RE cycle in a Pn(III)/Pn(V) manifold is considered yet challenging, the Bi(I)/Bi(III) redox cycle has enabled a catalytic cycle of such sequence by a main group complex, which is conventionally confined to TM catalysis.

It is also shown that this *N*,*C*,*N*-chelated Bi platform for a Bi(I)/Bi(III) redox cycle is sufficiently robust to be applied in an electrocatalytic hydrogen evolution reaction (HER). The bismuthinidene **24** was in situ generated from the parent Bi(III) by electrochemical reduction, which oxidatively evolves hydrogen in the presence of acetic acid.^62^

## 3 High-Valent Bi(III)/Bi(V) Catalysis

**Figure 9:**
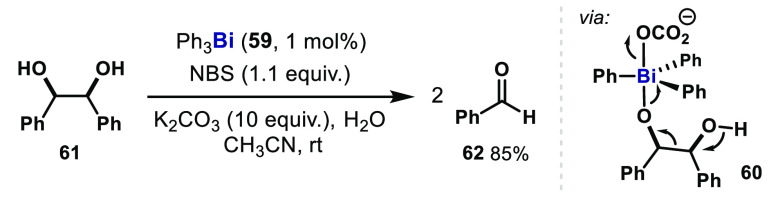
Ph_3_Bi-catalyzed α-glycol cleavage via Bi(V) intermediates.

Seminal work that capitalize upon the redox properties of organobismuth(III) in catalysis was reported by Barton and Motherwell in 1981, in which a Ph_3_Bi catalyst (**59**) was engaged with *N*-bromosuccinimide (NBS) as a stoichiometric oxidant for the C–C scission in 1,2-diols (Figure 9).^63^ Similar to the previously reported oxidative reactions by Bi(V) reagents,^26^ the stochiometric oxidation of an α-glycol **61** by triphenylbismuth carbonate resulted in the formation of benzaldehyde (**62**) presumably via a Bi(V) intermediate **60**. In this reaction, Ph_3_Bi was formed in quantitative yield (**59**), and thus, the catalytic reactions were readily achieved by slow addition of NBS for regeneration of Bi(V) species. Related oxidative reactivity has also been reported using Ph_3_Sb.^20^ However, in these examples, the organometallic compounds are mere oxidants and the Bi–C bonds remain intact throughout the catalysis.

**Figure 10:**
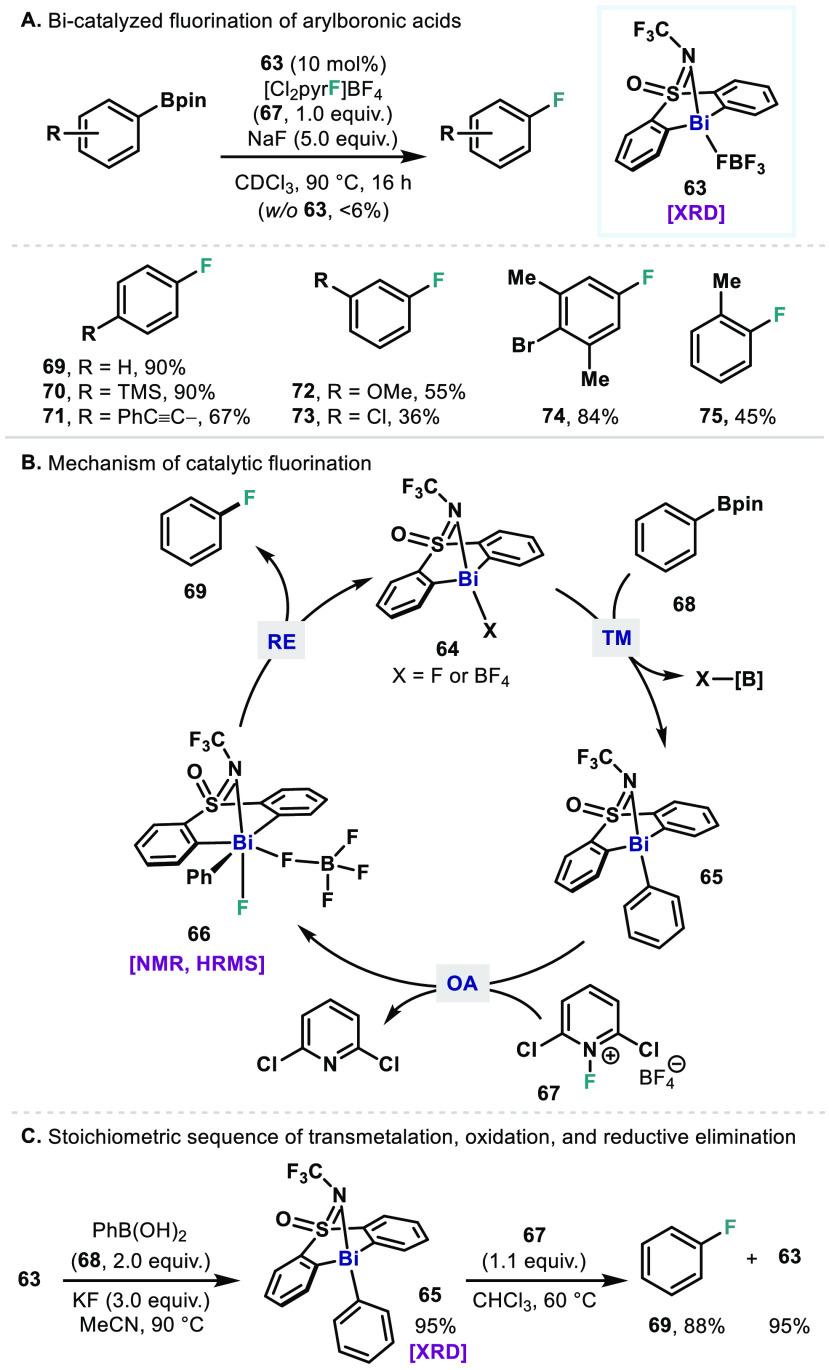
(A) Bismuth-catalyzed fluorination of aryl boronic esters. (B) Proposed mechanism of fluorination via a Bi(III)/Bi(V) cycle. (C) Stoichiometric sequence of elementary steps: transmetalation and oxidation–reductive elimination.

Stoichiometric examples where the aryl ligands on organobismuth(V) complexes are engaged in reactivity can be found in the arylation of organic compounds through oxidative ligand coupling or formal reductive elimination.^28,64^ In order to advance these stoichiometric reactions to catalysis, the oxidation step (Bi(III) → Bi(V)) should be compatible with other elementary steps, and after ligand coupling, the commonly reactive aryl group must be reincorporated. In 2020, a demonstration of bismuth redox catalysis via canonical organometallic elementary steps in a Bi(III)/Bi(V) platform was described in the catalytic fluorination of aryl boronic esters (Figure 10A).^65^ A bismuth catalyst (**63**) supported by a dianionic bis-aryl sulfoximine ligand is used to enable Bi(III)/Bi(V) redox cycling, where the NCF_3_ coordinates to the Bi center as a weak ligand. With this ligand platform, Bi(III) catalyst **63** (10 mol %) drives fluorination of aryl boronic esters in the presence of 2,6-dichloro-1-fluoropyridinium tetrafluoroborate (**67**, 1 equiv) and sodium fluoride (5 equiv). Boronic esters with *para*-substitution are generally well-tolerated (**70**, **71**). Those with *meta*- or *ortho*-substitution are amenable but relatively more challenging (**72**–**75**). Notably, the reaction did not proceed in the absence of the bismuth catalyst.^66^

The catalytic Bi(III)/Bi(V) redox cycle is proposed for this fluorination on the basis of stoichiometric reactivity and identification of key intermediates (Figure 10B). It is proposed that the Bi(III) catalyst **63** first undergoes transmetalation with an aryl boronic ester to generate phenylbismuthine **65**. The complex **65** is then oxidized by fluoropyridinium reagent **67** to afford a high-valent Bi(V) species **66** that is stabilized by coordination of the pendant NCF_3_ moiety. The resulting Bi(V) species **66** undergoes reductive elimination of fluorobenzene **69** to regenerate Bi(III) species **64**, enabling Bi(III)/Bi(V) redox cycling. These elementary steps, including transmetalation, oxidative addition, and reductive elimination, have all been confirmed to proceed stoichiometrically (Figure 10C). The transmetalation product **65** was crystallographically characterized, and the following oxidation step yielded the fluorination product **69** and regenerated the Bi(III) catalyst **63**. The Bi(V) intermediate **66** was independently synthesized and shown to undergo reductive elimination of C–F bond to give **69**. Indeed, they are reminiscent of those in classic TM-catalyzed cross-coupling reactions. However, this Bi(III)/Bi(V) redox catalysis does not merely mimic TM catalysis but enables a catalytic reaction that is considered to be challenging with TMs.^67−70^ This chemistry exhibits the potential of bismuth redox catalysis to unveil new reactions unique to bismuth.

In addition, it is interesting that the Bi(III)/Bi(V) manifold operates fluorination whereas the Bi(I)/Bi(III) manifold can drive defluorination of polyfluoroarenes. These two catalytic reactions explicitly exemplify that both catalytic oxidative and reductive chemistry can be achieved by employing relevant Bi redox couples.

**Figure 11:**
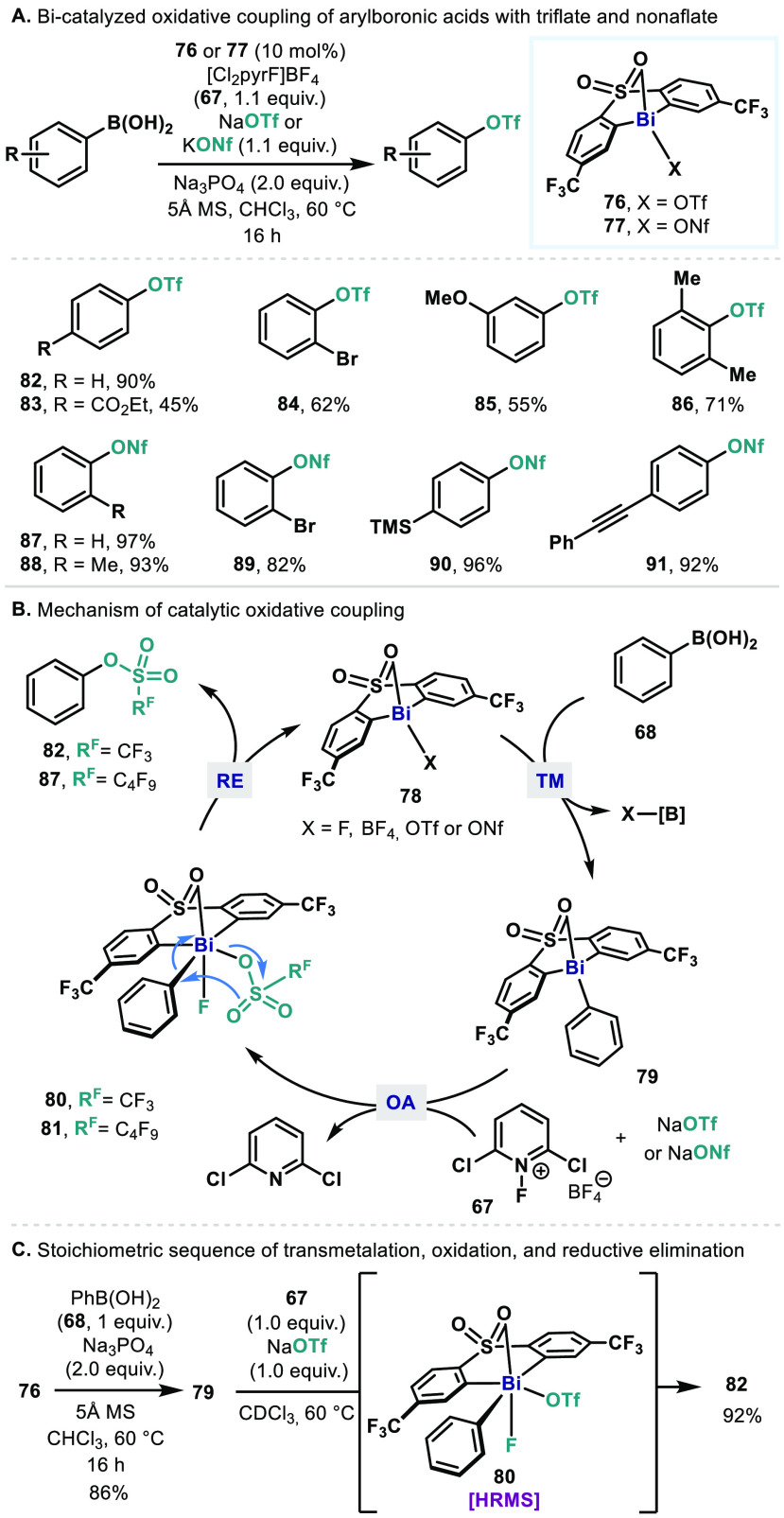
(A) Bismuth-catalyzed triflation and nonaflation of arylboronic acids. (B) Proposed mechanism via a Bi(III)/Bi(V) cycle. (C) Validation of elementary steps including transmetalation and reductive elimination via a Bi(V) intermediate.

The tethered bis-aryl ligand platform bearing a pendant neutral ligand was further exploited in an oxidative coupling of arylboronic acids with perfluoroalkyl sulfonate salts (Figure 11A).^71^ A related stoichiometric reaction was previously reported by Mukaiyama, in which a stoichiometric oxidative coupling between phenylbismuthine species and an excess of triflic acid in the presence of *m*-chloroperoxybenzoic acid (*m*CPBA) gives phenyl triflate (**82**) in 29% yield.^28f^ This reductive elimination type reaction was advanced into Bi-catalyzed triflation and nonaflation of aryl boronic acids using the corresponding perfluoroalkyl sulfonate salts. In this catalysis, a modified Bi(III) catalyst **76** ligated with bis(*m*-trifluoromethyl-phenyl) sulfone is used. A more electron-deficient backbone is introduced to render the Bi(III) a better leaving group during C–O bond formation. Indeed, Bi(III) catalysts **76** and **77** (10 mol %) drive triflation and nonaflation of aryl boronic acids, respectively, in the presence of a perfluoroalkyl sulfonate salt (NaOTf or KONf, 1.1 equiv), an oxidant **67** (1.1 equiv), and Na_3_PO_4_ (2 equiv). This method accommodates aryl boronic acids with various types of substitution (**82**–**91**).

Analogous to the fluorination catalyzed by **63**, the Bi(III) catalyst (**76** or **77**) is proposed to undergo transmetalation with aryl boronic acids (Figure 11B). Subsequent oxidation of the resulting phenylbismuthine **79** by oxidant **67** generates a putative high-valent Bi(V) species (**80** or **81**), which can be detected by HRMS in the corresponding stoichiometric reaction with NaOTf (Figure 11C). The Bi(V) intermediate (**80** or **81**) then undergoes reductive elimination of the corresponding aryl triflate **82** or aryl nonaflate **87** with the regeneration of Bi(III) species (**78**). DFT calculations suggest that ligand coupling occurs via a five-membered transition state in which two oxygens of triflate are involved. This transition state is shown to be favored over a three-membered transition state via which reductive elimination from transition metals typically occurs (Δ*G*_5-mem,calc_^⧧^ = 22.9 kcal/mol Δ*G*_3-mem,calc_^⧧^ = 24.9 kcal/mol).

Given that triflate and nonaflate are highly electronegative, poorly nucleophilic, and weakly coordinating,^72^ C–O bond formation is inherently challenging using TM complexes and a few chemistry is known for triflation steps by transition metals.^73^ As revealed in fluorination catalysis, this oxidative coupling reaction via a Bi(III)/Bi(V) cycle has also enabled new transformations that are relatively challenging in TM catalysis by taking advantage of the unique reactivity of bismuth complexes.

## 4 Redox-Neutral Bi(III) Catalysis

**Figure 12:**
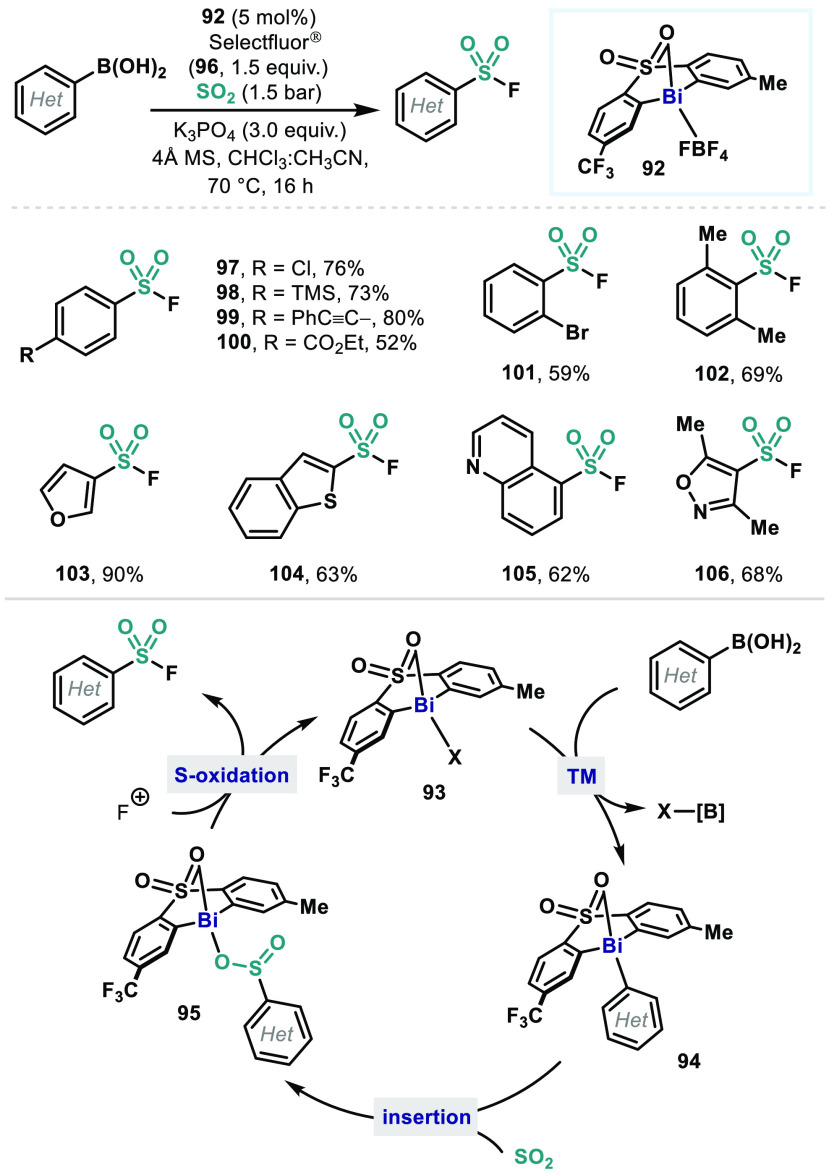
Bismuth-catalyzed synthesis of (hetero)aryl sulfonyl fluorides and proposed mechanism via redox-neutral elementary organometallic steps.

While non-redox Bi catalysis has been dominated by Lewis acid catalyzed transformations, redox-neutral Bi(III) catalysis in which a Bi(III) catalyst does not act as a mere Lewis acid has been recently demonstrated in a catalytic synthesis of (hetero)aryl sulfonyl fluorides from (hetero)aryl boronic acids using sulfur dioxide (SO_2_) and Selectfluor (**96**) (Figure 12).^77^ A Bi(III) catalyst **92** was found to be an optimal catalyst for this reaction, which was discovered through modification of the diarylsulfone ligands that were used in Bi(III)/Bi(V) redox catalysis. Analogous to the fluorination and the triflation catalysis (section 3), the catalyst **92** readily undergoes transmetallation with a (hetero)aryl boronic acid to generate (hetero)arylbismuthine **94**. Although stoichiometric reactivities of both Bi(III)–C^78^ and Bi(V)–C^79^ toward SO_2_ or its surrogates have been known, it was found that Bi(III) intermediate **94** is an active species for the insertion of SO_2_ into the Bi–C bond which gives a diarylbismuth sulfinate **95**. Upon oxidation by electrophilic fluorine source, complex **95** then affords the corresponding (hetero)aryl sulfonyl fluoride with regeneration of catalytically active Bi(III) species **93**. In this catalytic reaction, it was demonstrated that the oxidant (**96**) does not oxidize the Bi center but the S(IV) to form the S–F bond. Thus, unlike aforementioned Bi(III)/Bi(V) redox catalysis, this reaction does not involve Bi(V) intermediates, achieving a redox-neutral Bi(III) catalysis with an insertion step. This method can tolerate various functional groups (**97**–**102**). In addition, by using a milder oxidant, *N*-fluorobenzenesulfonimide (NFSI), instead of **96**, heteroaryl boronic acids can be tolerated (**103**–**106**), which has been challenging for Bi catalysis using strong oxidants. Compared to related two-step sequence TM-catalyzed reactions,^80^ Bi catalysis provides a convenient and competent synthetic method for sulfonyl fluorides with high functional group compatibility.

## 5 Conclusions and Future Outlook

In this Perspective, we provided an overview of recent efforts to leverage the Bi(II)/Bi(III), Bi(I)/Bi(III), and Bi(III)/Bi(V) redox couples for discovery of new reactivity and development of new main group catalysis. It is clear that the field of bismuth redox catalysis is still in its infancy, yet it has provided compelling evidence that new applications are awaiting in the area of organic synthesis, which could certainly reach beyond those catalyzed by transition metals. Looking ahead, we anticipate numerous opportunities for discovery in bismuth redox catalysis:

(1) While other elementary steps (e.g., OA, LM, RE) comprising TM catalysis are analogously realized in Bi catalysis, combinations with migratory insertion-type elementary steps are relatively less explored.^28i,49b,77,74^ Incorporating this type of insertion step into a catalytic cycle would enable new transformations, using small molecules as building blocks.
(2) Compared to organo–Bi(I), −Bi(III), and −Bi(V) compounds, open-shell −Bi(0), −Bi(II), and −Bi(IV) species are much less explored or unknown.^75^ These species might provide further opportunities to unveil new catalytic cycles and reaction pathways. For example, dual photoredox/bismuth catalysis or combined electrochemistry with bismuth, could be envisioned to precisely control oxidation or reduction of bismuth complexes and ultimately enable selective or challenging transformations.
(3) Combined with diverse redox catalysis and the robust nature of Bi complexes, development of molecular Bi-based electrocatalysts is feasible, which could provide better understanding in heterogeneous electrocatalysis at the molecular level.^62^
(4) Given the orthogonal or complementary reactivity of Bi catalysts, Bi redox cycles could be merged with TM catalysis to achieve challenging transformations via dual Bi/TM catalysis.
(5) In more-complex settings, stereoselective redox transformations or bioconjugation reactions could be devised for organobismuth complexes, because of the facile introduction of chiral elements around its Bi center, and their benign nature and stability in aqueous solutions, respectively.
(6) Finally, dinuclear or multinuclear complexes with mixed oxidation states would also allow one to discover new redox chemistry.^76^

We hope that this Perspective will draw more attention to the infant field of redox catalysis using bismuth, provide guiding principles for new advancements in catalysis and synthesis, and promote realization of the full potential of such a new area of expertise.
